# SOCIAL AND CULTURAL FACTORS FOR HOSPICE CARE OUTCOMES: PERSPECTIVES OF PATIENTS, CAREGIVERS, AND PROVIDERS

**DOI:** 10.1093/geroni/igz038.2910

**Published:** 2019-11-08

**Authors:** David Russell, Dawon Baik, Lizeyka Jordan, Frances Dooley, Ruth M Masterson Creber

**Affiliations:** 1 Visiting Nurse Service of New York, New York, New York, United States; 2 Columbia University, New York, New York, United States; 3 Weill cornell Medical College, New York, New York, United States

## Abstract

Use of hospice services in the U.S. has grown to cover an expanding number of patients with varying conditions and demographic characteristics. Notably, hospice agencies increasingly serve patients with diverse socio-cultural and linguistic backgrounds. Limited research has explored how these factors act to influence the course and outcomes of hospice care, or their role in shaping race/ethnic and socioeconomic disparities in burdensome outcomes like acute hospitalization. This presentation uses the theoretical lens of Cultural Health Capital to explore how socio-cultural factors affect patient-provider interactions within the home hospice setting. Qualitative interviews were conducted with both providers (N=32) and patients/caregivers (N=7) at a large not-for-profit hospice agency in New York City. Themes identified from these interviews included prior knowledge and familiarity with hospice, family dynamics and social support, and linguistic and cultural barriers to care. Findings indicate the need for greater attention to socio-cultural influences on interactional dynamics within home hospice.

